# Polycyclic Aromatic Hydrocarbons Affect Rheumatoid Arthritis Pathogenesis *via* Aryl Hydrocarbon Receptor

**DOI:** 10.3389/fimmu.2022.797815

**Published:** 2022-03-22

**Authors:** Xiaoyu Xi, Qinbin Ye, Danping Fan, Xiaoxue Cao, Qiong Wang, Xing Wang, Mengxiao Zhang, Yuan Xu, Cheng Xiao

**Affiliations:** ^1^ School of Traditional Chinese Medicine, Beijing University of Chinese Medicine, Beijing, China; ^2^ Institute of Clinical Medicine, China-Japan Friendship Hospital, Beijing, China; ^3^ Graduate School of Peking Union Medical College, Chinese Academy of Medical Sciences/Peking Union Medical College, Beijing, China; ^4^ Department of Traditional Chinese Medicine (TCM) Rheumatology, China-Japan Friendship Hospital, Beijing, China; ^5^ Department of Emergency, China-Japan Friendship Hospital, Beijing, China

**Keywords:** rheumatoid arthritis, polycyclic aromatic hydrocarbons, particulate matter, aryl hydrocarbon receptor, aryl hydrocarbon receptor repressor

## Abstract

Rheumatoid arthritis (RA), the most common autoimmune disease, is characterized by symmetrical synovial inflammation of multiple joints with the infiltration of pro-inflammatory immune cells and increased cytokines (CKs) levels. In the past few years, numerous studies have indicated that several factors could affect RA, such as mutations in susceptibility genes, epigenetic modifications, age, and race. Recently, environmental factors, particularly polycyclic aromatic hydrocarbons (PAHs), have attracted increasing attention in RA pathogenesis. Therefore, exploring the specific mechanisms of PAHs in RA is vitally critical. In this review, we summarize the recent progress in understanding the mechanisms of PAHs and aryl hydrocarbon receptors (AHRs) in RA. Additionally, the development of therapeutic drugs that target AHR is also reviewed. Finally, we discuss the challenges and perspectives on AHR application in the future.

**Graphical Abstract:**
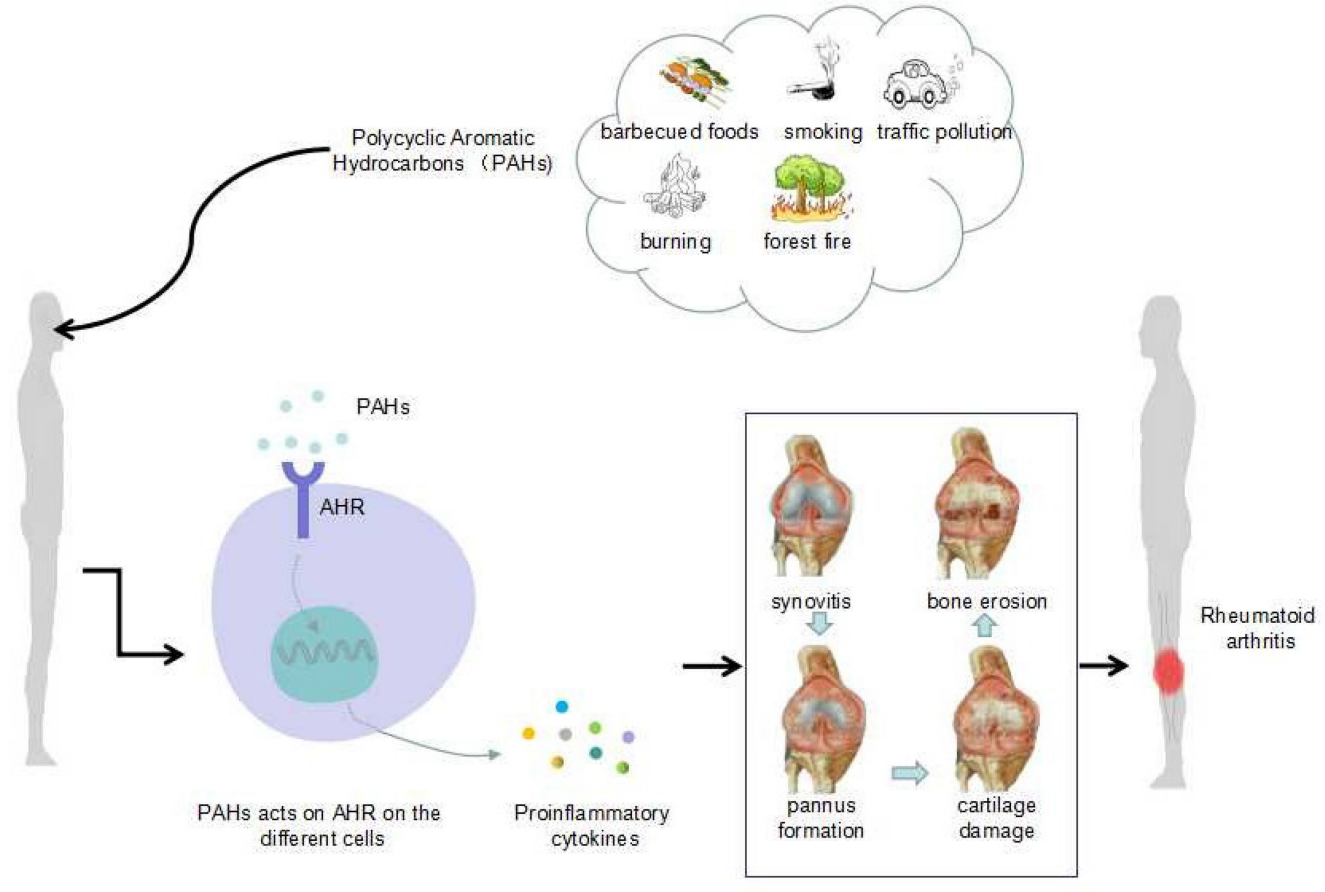


## Highlights

- We explored the close relationship between PAHs and RA and summarized previous studies.
- PAHs affect the pathogenesis of RA through the AHR pathway.
- We explored the treatment strategies for RA and current novel strategies based on the AHR pathway.

## 1 Introduction

Rheumatoid arthritis (RA) is the most common autoimmune disease characterized by changes in the local inflammatory environment, swollen joints and pain; eventually leads to joint dysfunction or contributes to disability. In recent years, the prevalence and incidence rates of autoimmune diseases such as RA have risen rapidly worldwide (1, 2). Autoimmune diseases are one of the most common diseases in the United States, affecting approximately 8% of the population (3), and they are one of the leading causes of death among young and middle-aged women. Some of the affected individuals also have anxiety or depression because of the failure of medication, substantially affecting quality of life. Additionally, in the late stage of RA, respiratory, circulatory and tumor diseases can occur, and the probability of death is further increased. The high incidence and serious consequences of RA have attracted extensive attention, and the depth of research on RA has been increasing.

At present, the etiology and pathophysiology of RA are mainly related to genetic and environmental factors; genetic inheritance accounts for 30% of all autoimmune diseases, while environmental factors, which account for a large proportion of cases, are the primary factors. Genetic factors mainly include human leukocyte antigen-DRB1 (HLA-DRB1), and the main environmental factor is polycyclic aromatic hydrocarbons (PAHs) (4, 5). PAHs are ubiquitous air pollutants formed by the burning and decomposition of coal, oil, garbage, natural gas and other substances. PAHs are closely related to various diseases. In addition to RA, cardiovascular, cerebrovascular, and respiratory system diseases also exist, and PAHs exposure can also lead to metabolic diseases such as diabetes (6) and carcinogenic effects (7).

Many studies have proven that PAHs play a crucial role in the development of RA through various ways (4, 5), including inducing the changes of immune cells and corresponding cytokines (CKs). Immunoregulatory cells and CKs play a vital role in immune homeostasis and the pathogenesis of RA. Previous studies have shown changes in inflammatory environments, such as the infiltration of inflammatory cells and CKs in the local joint RA patients. Several studies have reported the number and function of T cells and B cells, and the corresponding CKs are closely related to RA (8). In addition to immune cells, changes in autoantibodies are also common markers in RA, mainly including rheumatoid factor (RF) and anti-citrullinated protein antibodies (ACPAs) (9–11), which is mainly achieved by activating B cells. These complex alterations are primarily caused *via* the action of aromatic hydrocarbon receptor (AHR). But assessing the detailed impact of PAHs on RA is a massive challenge because through there are many basic studies on the relationship between PAHs and RA, the specific mechanism of PAHs and AHR in the pathological mechanism of RA remains unclear.

In this review, we mainly summarized recent developments of PAHs in RA studies and the RA mechanism *via* PAHs of environmental pollution and AHR signaling pathway. Additionally, we discussed the therapeutic potential and application prospects of targeting AHR in RA, providing crucial information for discovering novel and effective RA treatments.

## 2 Sources and Effects of PAHs in RA

### 2.1 Environmental Pollution and PAHs

The factors causing environmental pollution mainly include two major aspects: factors derived from human influence, such as those derived from barbecued foods, smoking, urban traffic pollution, the burning of oil and the random burning of garbage, and factors derived from natural causes, such as forest fires. Thus, mixed ambient pollutants are produced, such as sulfur dioxide, carbon monoxide, nitrogen dioxide and particulate matter (PM). PM is further classified into several types according to the particle size: PM 10 (<10 μm in diameter), PM 2.5 (<2.5 μm in diameter), PM 1.0 (<1.0 μm in diameter), PM 0.1 (<0.1 μm in diameter) and ultrafine particles (UFPs) (in order from largest to smallest).

Abundant PAHs are found in barbecued foods, and cigarette smoke also contains many PAHs. A previous study in Bangladesh found that people who were overexposed to urban traffic pollution were exposed to high levels of PAHs (12). This finding suggests that in Dhaka, urban residents who are exposed to traffic pollution are at a higher risk of exposure to carcinogenic PAHs. Not surprisingly, excessive amounts of PAHs are also found in waste incinerators and decomposition products of oil burning. Generally, PAHs are the major components in environmental particulate pollutants and have adverse health effects (that is, they are hazardous to health). Additionally, among the PM types, those that pose the main threat to human health are UFPs (13), which are too small for airway mucous cilia and alveolar macrophages to eliminate and therefore become deposited in the lungs.

Although extensive studies have proven that an increased risk of RA is closely related to environmental pollution (14–18), the exact mechanism between RA and environmental pollution is poorly clear.

### 2.2 PAHs and RA

#### 2.2.1 General Overview of PAHs

PAHs are the most common pollutants in the environment and are a group of organic compounds comprising two or more aromatic rings in different configurations that are mainly derived from the burning of coal and natural gas, the indiscriminate burning of garbage and other organic compounds such as tobacco and barbecued foods. A few PAHs are used as pharmaceuticals and pesticides and in plastics, while others are included in the asphalt used for road construction. More than 100 PAHs have been identified in the environment, and sixteen of them are listed as primary pollutants by the United States Environmental Protection Agency. PAHs exist primarily as mixtures instead of single compounds. Additionally, they tend to become deposited in the human body and remain within the food chain because of their lipophilic properties (19).

Halogenation of PAHs gives them metabolic and environmental stability. Generally, PAHs are agonists of and have a high affinity for AHR, leading to halogenation of PAHs side rings; PAHs also show distinct affinity for and are easily metabolized by CYP enzymes (20). However, several PAHs have been demonstrated to inhibit the generation of CYP enzymes, inhibiting their metabolism and that of other organic components present in mixtures (21, 22).

Recently, the effects of PAHs on human health have been widely studied, and many experimental studies have recently focused on the link between PAHs and RA (4, 5). Novel studies have demonstrated that high levels of PAHs in urine are significantly related to a high incidence of RA, implying that PAHs exposure may increase the incidence of RA (23, 24).

#### 2.2.2 General Biology of AHR

AHR is a ligand-dependent transcription factor that plays a crucial role in regulating the differentiation, activation and apoptosis of various cells in RA. AHR is best known for its capacities to mediate the toxicity of dioxin and is normally present in the immune cell cytoplasm as a part of a complex of multiple proteins, primarily comprising heat shock protein (HSP) 90, P23, which is a scaffold protein, and activated AHR. According to molecular cloning studies, AHR contains a basic helix-loop-helix (bHLH) domain, which is similar to that found in DNA-binding proteins (25, 26). Additionally, AHR contains a PER-ARNT-SIM (PAS) homology domain, including a PAS A and PAS B domain, similarly found in other regulators of cell and organism responses to the environment (27, 28). Interestingly, the binding of ligands to AHR mainly occurs *via* the PAS B domain.

Activation of the AHR signaling pathway depends on the ligands, including endogenous and exogenous ligands. These ligands can be present in the environment or produced by metabolism. AHR-mediated signaling pathways may be essential for the immune regulatory response, and previous studies have revealed that AHR signaling not only affects innate immunity but also participates in adaptive immunity in the development of diseases, such as RA.

Recent studies have also suggested that abnormal activation of AHR signaling pathways may be associated with autoimmune diseases, such as RA, multiple sclerosis (MS), systemic lupus erythematosus (SLE), autoimmune uveitis (AU), myasthenia gravis (MG) and Bechet’s disease (BD). Importantly, regulating AHR’s involvement in autoimmune diseases is multidimensional with 3-MC or other ligands, which can disrupt the balance between immune cells, significantly shift the Th1/Th2 balance in favor of a Th1 response, modulate the differentiation of regulatory T (Treg) cells, and increase the proliferation and differentiation of Th17 cells. Not surprisingly, several studies have shown that AHR has opposite effects on Treg cell and Th17 cell differentiation (29, 30). Furthermore, AHR can regulate the activity of dendritic cells (DCs), natural killer (NK) cells and macrophages. These changes in immune cells eventually lead to and exacerbate autoimmune diseases.

More novel ideas have been confirmed, in addition to Th17 cells being activated in a manner dependent on the AHR pathway, the same is true for Th1, Th2 and Treg cells. In recent years, AHR has been studied in various aspects of immunology, mainly focusing on its effect and regulation of T cell differentiation, maturation and function. AHR is highly expressed in several CD4+ cells; the highest expression is observed on Th17, and FoxP3+ Treg cells, followed by Th1 and Th2 cells (31, 32).

#### 2.2.3 Signal Transduction Pathway of AHR

**Figure 1:**
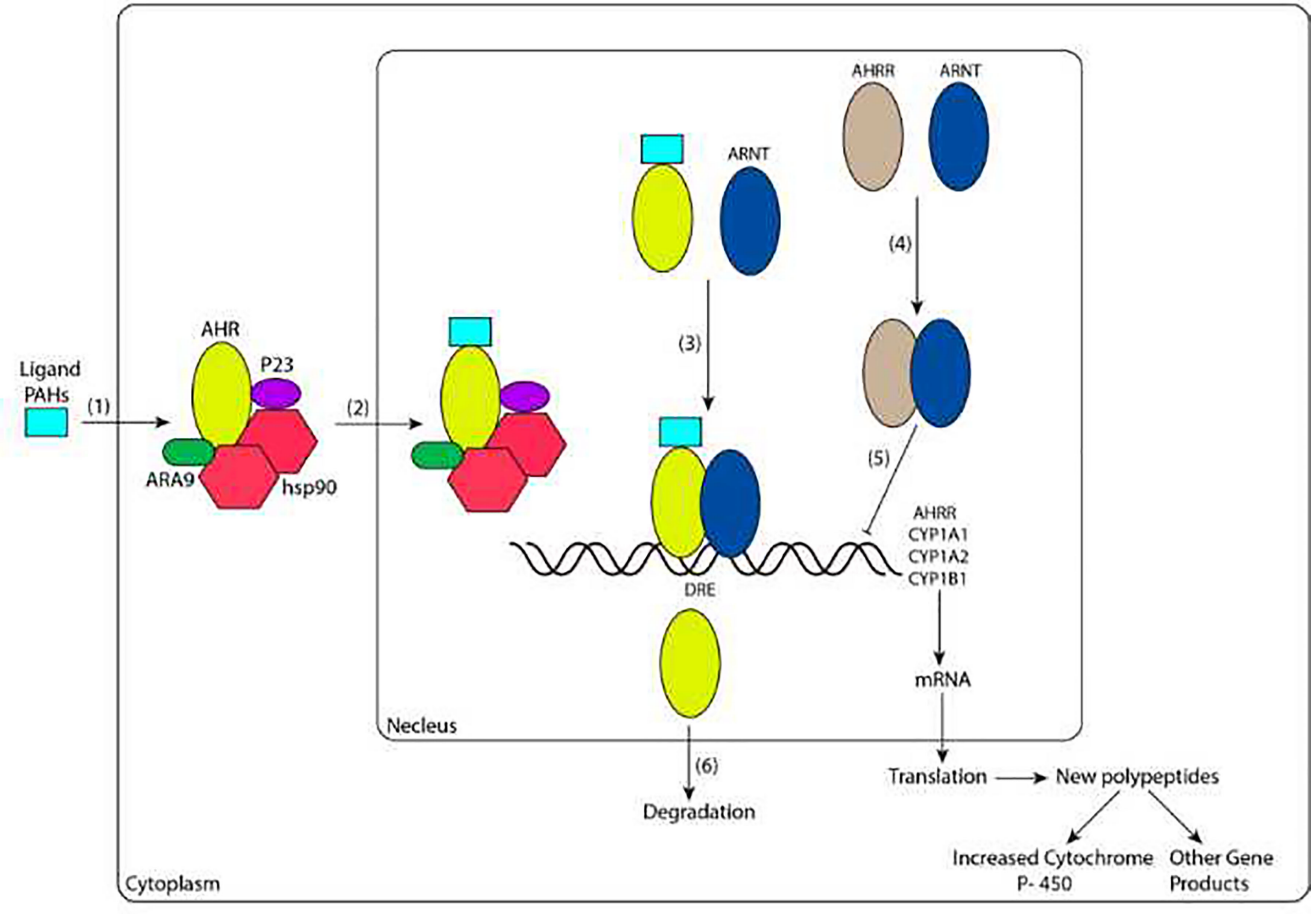
Ligand-dependent signal transduction pathways of AHR activation. (1) The ligand diffuses into cells and binds to the AHR complex in the cytoplasm. (2) The ligand-bound receptor complex translocates into the nucleus. (3) The heterodimerization of AHR and ARNT enhances the binding ability with DREs. (4) AHRR binds to ARNT. (5) AHRR and ARNT dimers repress AHR transcriptional activity. (6) AHR is exported to the cytoplasm by nuclear exportation and degraded. CYP1A1, CYP1A2, and CYP1B1 are genes encoding the phase I enzyme P450. AHR, AHRR and ARNT are basic helix-loop-helix-PER-ARNT-SIM (bHLH-PAS) proteins, and AHRR and AHR have high sequence similarity. PAHs, polycyclic aromatic hydrocarbons; AHR, aromatic hydrocarbon receptor; hsp90, heat shock protein 90; ARNT, aromatic hydrocarbon receptor nuclear translocation; AHRR, aryl hydrocarbon receptor repressor; DRE, dioxin response element; CYP, cytochrome P.

Numerous studies have indicated that AHR plays a critical role in immunomodulation (33, 34). After activation of AHR, intracellular signaling occurs through genomic and/or nongenomic pathways **(** **Figure 1** **)**. In this review, we mainly discuss the activation pathway of AHR from the perspective of genomic pathways.

PAHs are ubiquitous pollutants and always exist in the environment as a mixture. Overexposure to PAHs usually activates AHR in immune cells, such as Th1 and Th17 cells, resulting in the promotion of inflammatory CKs production and an increased RA incidence. Additionally, PAHs inhibit the activation of AHR in Th2 and Treg cells and weaken the ability to produce interleukin-10 (IL-10), transforming growth factor-β (TGF-β) and IL-4 to further promote the occurrence of RA (31, 35, 36).

Once agonists (ligands such as PAHs) reach the immunomodulatory cell and bind to the PAS domain of AHR, a conformational change of AHR is induced. This change alters the binding of AHR with other chaperones and leads to the nuclear localization signal being fully exposed. Thus, the ligand-AHR complex is transferred to the nucleus. When the ligand-AHR complex is disassociated from the complex containing HSP90, ARA9 and P23, it forms a heterodimer with another protein, bHLH-PAS, also known as AHR nuclear translocation (ARNT). In the nucleus, the interaction between AHR and ARNT (which forms AHR/ARNT heterodimers) increases their ability to bind to specific sequences of enhancers near the target gene promoter, known as dioxin response elements (DREs) (37, 38) ; this effect leads to the changing of downstream several genes, among these genes, the most well-studied include cytochrome P (CYP)1A1, CYP1A2, CYP1B1, and aryl hydrocarbon receptor repressor (AHRR), the former three of which encode a phase I enzyme, a heme-mercaptan protein and a key adaptor to metabolic reactions, known as CYP. CYP is involved in the metabolism of endogenous substances and exogenous substances, including drugs and environmental compounds. Additionally, the CYP enzyme is mainly located in the endoplasmic reticulum, where it is responsible for catalyzing the first stage of exogenous biological oxidation transformation (39) and regulating the activation, differentiation, and apoptosis of various cells (40). This biotransformation reduces the toxicity of compounds, but intermediates can be produced in the case of PAHs (41).

**Figure 2:**
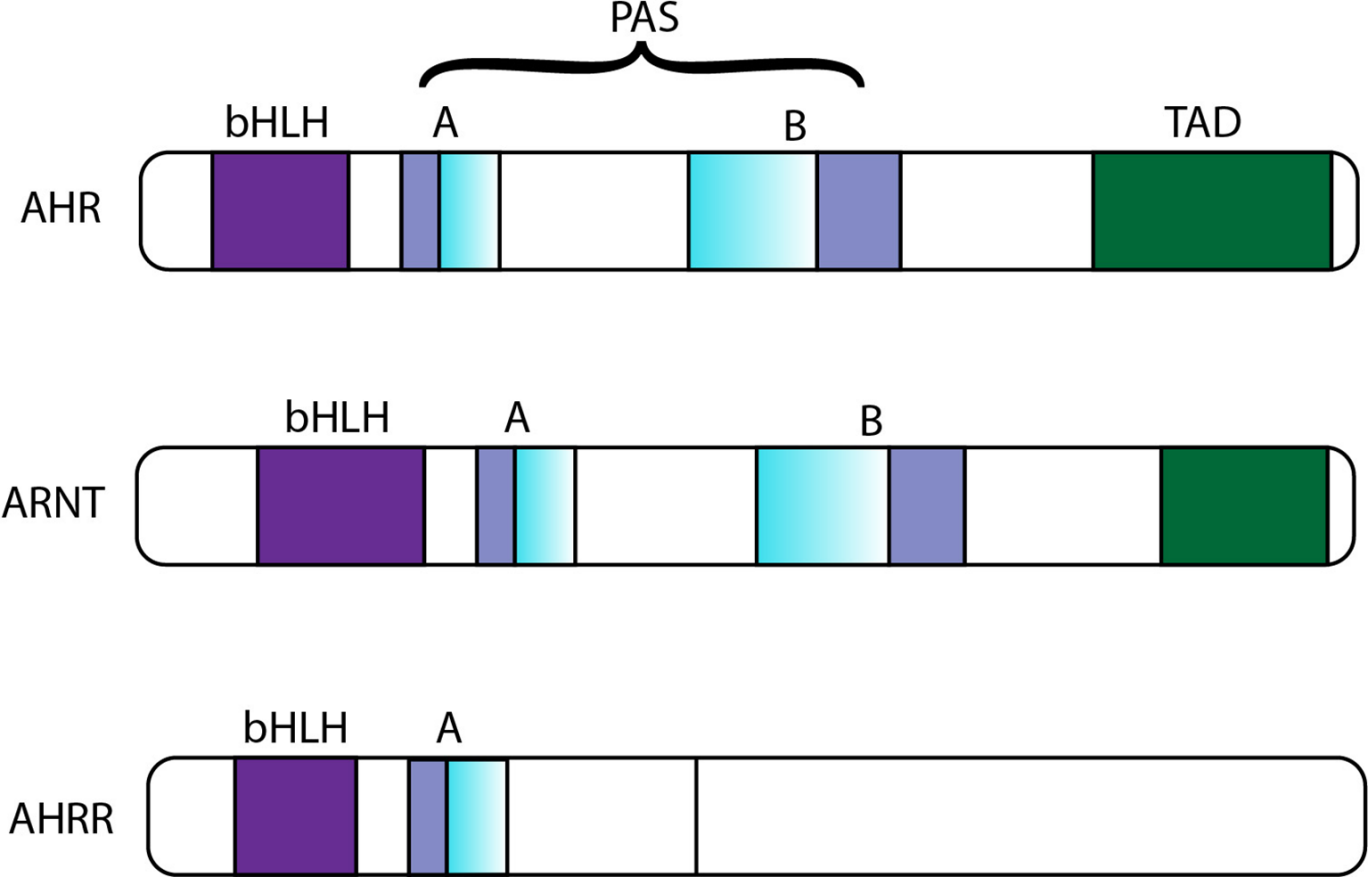
Sequence comparison of AHR, ARNT and AHRR. bHLH, basic helix-loop-helix; PAS, PER-ARNT-SIM; TAD, transcriptional activation domain; AHR, aromatic hydrocarbon receptor; ARNT, aromatic hydrocarbon receptor nuclear translocation; AHRR, aryl hydrocarbon receptor repressor.

In addition to positive activation of AHR, AHR is also negatively regulated. First, with ligand-induced activation and nuclear output (42, 43), AHR is degraded *via* the 26S proteasome (44, 45). The second mechanism of reduced activity of the AHR-ARNT complex is the upregulation of a transcriptional repressor called AHRR (46). AHRR is also a bHLH-PAS protein with a sequence with high similarity to the AHR and ARNT sequences **(** **Figure 2** **)**; specifically, AHR, AHRR and ARNT are expressed in most cells and tissues, and they show high similarity in their structures **(** **Figure 2** **)**. The three proteins are members of the bHLH-PAS family of transcription factors. The absence of the PAS B domain in the AHRR structure, which is vital for the ligand binding of AHR, contributes to the negative modulation of AHR by AHRR. Additionally, the carboxyl termini of AHR and ARNT include transcriptional activation domains (TADs). The TADs of AHR contain numerous independent active subdomains, while the TAD of ARNT is simpler **(** **Figure 2** **)**.

AHRR inhibits the transcriptional activity of AHR mainly by binding to ARNT, and the interaction between ARNT and AHRR inhibits the binding of compounds with DREs (46). Under normal conditions, positive and negative feedback is balanced, however, in the event of diseases, the balance between them is completely disturbed.

To some extent, CYP can promote the metabolism of PAHs *in vivo* to alleviate the occurrence of diseases, but PAHs exposure can inhibit the activity of CYP and worsen diseases. Several PAHs have been revealed to inhibit the activity of CYP enzymes to alter their own metabolism (21, 47). Specifically, the inhibitory effect of PAHs on CYP enzymes suppresses the production of water-soluble metabolites that is not conducive to the excretion of PAHs. Subsequently, PAHs penetrate the intestinal epithelium and enter the lamina propria and immunomodulatory cells, contributing to the acceleration of autoimmune diseases like RA (48). In recent years, the attention at PAHs has been gradually increasing, while the attention directed at AHR has also increased. An up-to-date summary of the changes caused by activation of the AHR pathway on the levels of immune cells and CKs *in vivo* when PAHs exposure contributes to RA will be described below.

## 3 PAHs Stimulation-Induced Changes in RA

**Figure 3:**
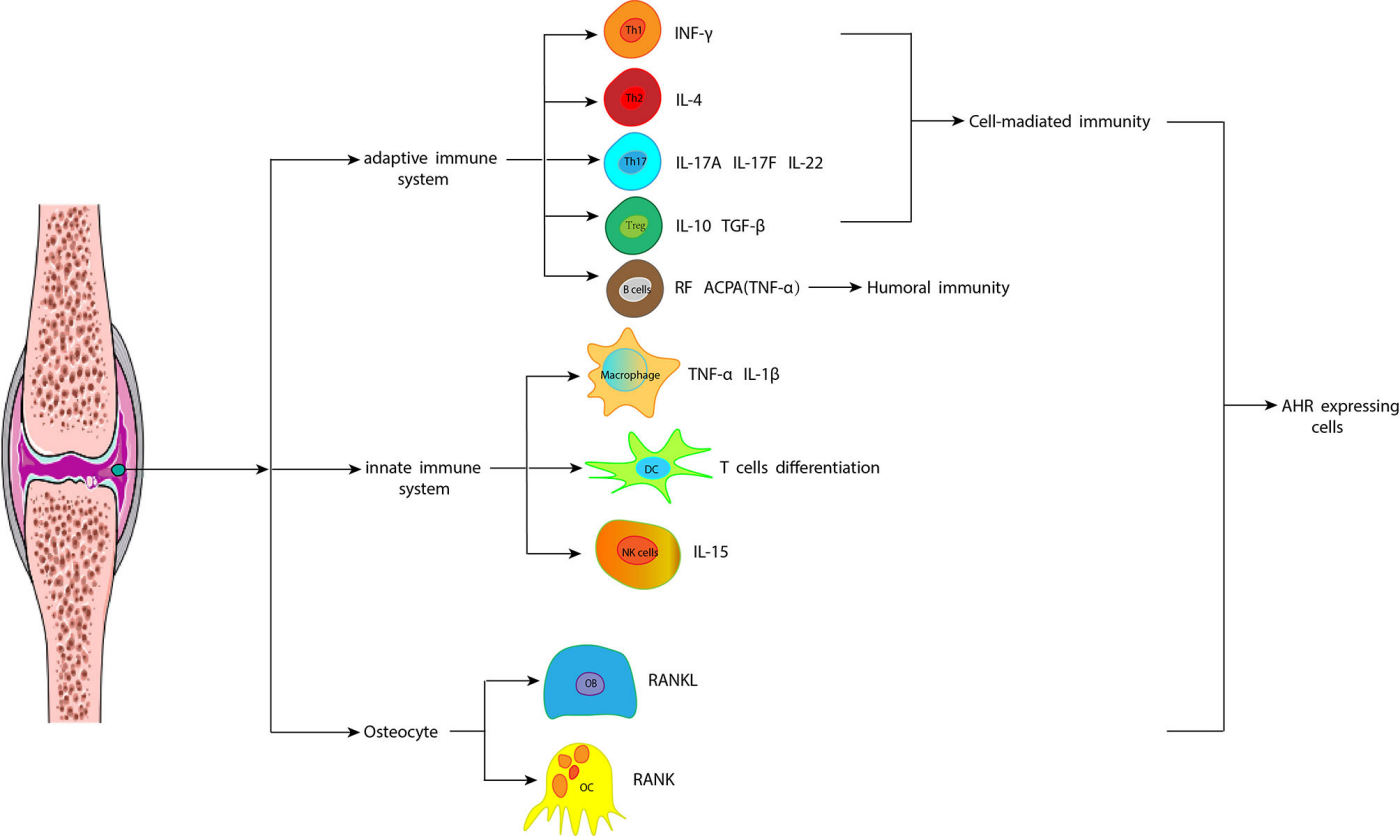
Different cells and their cytokines CKs play a key role in the development of RA through the AHR signaling pathway. The articular cavity is infiltrated by many inflammatory cells, which produce a mass of inflammatory mediators and induce an inflammatory cascade. These inflammatory cells include cells of the innate immune system, such as macrophages, dendritic cells, and natural killer cells, as well as T cells and B cells of the adaptive immune system. Additionally, reduced osteoblasts and increased osteoclasts lead to the destruction of subchondral bone, leading to the degeneration of articular cartilage. CKs, cytokines; RA, rheumatoid arthritis; AHR, aromatic hydrocarbon receptor; IFN-γ, interferon-γ; IL, interleukin; TGF-β: transforming growth factor-β; RF, rheumatoid factor; ACPA, anti-citrullinated protein antibody; TNF-α: tumor necrosis factor-α; NK, natural killer; OC, osteoclast; RANKL, receptor activator of NF-κB ligand; OB, osteoblast; RANK, receptor activator of NF-κB.

The effects of PAHs in RA mainly involve the changes of immunomodulatory cells and corresponding CKs **(** **Figure 3** **)**.

### 3.1 Cell Changes

PAHs are currently recognized as the main environmental pollutants causing RA. The pathogenesis of PAHs exposure-induced RA mainly involves regulating immune cells, and studies have found that PAHs exposure changes the distribution of T cell subtypes. The balance of immune cells is disordered, mainly represented by an increase in pro-inflammatory T cells and a decrease in Treg cells (49, 50). Eventually, the changes in downstream CKs corresponding to these cells are affected, such as promoting an increased number of pro-inflammatory cells and related CKs, inhibiting the production of anti-inflammatory cells and CKs.

Considering that PAHs are diverse, one experiment exposed mice to the 16 most common PAHs. Acenaphthylene (ANY), fluoranthene (FLT), benzo [a] pyrene (B[a]P), benzo [a] anthracene (B[a]A), benzo [b] fluoranthene (B[b]F), benzo [k] fluoranthene (B[k]F), dibenzo (a,h) anthracene (DBA), indeno [1,2,3-cd] pyrene (IPY), and benzo [ghi] perylene (BPE) significantly promoted the differentiation of Th17 and Th1 cells and B[a]P, B[a]A, B[b]F, and B[k]F induced more obvious inhibition of Treg cell differentiation; however, naphthalene (NAP), anthracene (ANT), phenanthrene (PHE), fluorene (FLU) and diphenyl had no significant effect on the differentiation of Th1, Th2, Th17 and Treg cells; interestingly, when AHR is activated by 1-pyrene (PYR), fluorene (FLU) plays a protective role in RA (24). It is speculated that different PAHs show different mechanism.

In addition to the CD4 T cells mentioned above, PAHs also affect the differentiation and maturation of B cells, DCs, macrophages, NK cells, osteoclasts (OCs) and osteoblasts (OBs).

#### 3.1.1 Th1 and Th2 Cells

Both Th1 and Th2 cells are CD4+ T cell subsets that participate in the development of autoimmune diseases but their mechanisms are different, the Th1 is pro-inflammatory cells, while Th2 is anti-inflammatory cells (51). Interferon-γ (IFN-γ) is the major CKs secreted by Th1 cells and can promote the differentiation and maturation of CD8+ cytotoxic T lymphocytes (CTLs), improving the phagocytosis ability of macrophages to kill parasitic pathogens in target cells and playing a role in cellular immunity. In contrast to the mechanism of Th1 cells, Th2 cells secrete IL-4, which mainly stimulates the proliferation and differentiation of B lymphocytes, producing antibodies such as IgE to clear extracellular pathogens within the normal range and mainly playing a role in humoral immunity. However, excessive IgE production can lead to immune hypersensitivity, as observed in asthma. Therefore, the balance between Th1 and Th2 cells plays a key role in the immune response, and imbalance of these cells leads to immune diseases (52), such as RA. A significant imbalance between Th1 and Th2 cells has been reported in collagen-induced arthritis (CIA) mice and RA patients (53).

As the receptor of PAHs, AHR normally exists within the cytoplasm in Th1 and Th2 cells (36). A study confirmed that when PAHs (3-MC) activate AHR, oxidative stress significantly activates nuclear factor kappaB (NF-κB), which stimulates the airway production of Th1 cells; thus, the balance between Th1 and Th2 cells is biased toward Th1 cells, while Th2 cell production and CKs secretion are inhibited (36), functions that are associated with decreased expression of GATA-3, a critical factor in Th2 cell differentiation, this study suggests that exposure to PAHs results in an imbalance of Th1/Th2 cells and the occurrence of RA and that the intermediary involvement of AHR is even more important. Another RA mouse model showed that the lack of AHR in T cells inhibited the development of arthritis (35).

#### 3.1.2 Th17 Cells

Th17 cells are a relatively newly discovered subset of CD4+ T cells. ROR-CT, a transcription factor expressed on the surface of Th17 cells (54), plays a crucial role in the control of extracellular pathogens and plays a critical role in human autoimmunity. Th17 cells secrete key pro-inflammatory CKs, such as IL-17A, IL-17F, and IL-22 (55). Studies have revealed that the upregulation of Th17 cells and relevant pro-inflammatory CKs is closely related to the formation and severity of RA (55).

PAHs are common exogenous AHR ligands, continuous overexposure to PAHs causes a series of pro-inflammatory changes in humans. Adequate experiments have shown that AHR plays a crucial role in the activation and proliferation of Th17 cells (31, 56). Additionally, PAHs in PM act directly on T cells through AHR-dependent and CYP-dependent pathways to promote Th17 cell differentiation and the immune response (49). AHR is expressed in the cytoplasm of Th17 cells (31); and when it is activated by PAHs in PM, the proliferation and differentiation of Th17 cells and their ability to produce pro-inflammatory CKs are enhanced during the development of RA (56). Notably, AHR is not involved in the initial stage of Th17 cell differentiation; instead, it is important for terminal differentiation.

Consistent with this view, the production of IL-17 by Th17 cells is significantly reduced and the IL-22 production is completely inhibited when AHR dificiency (57). Published results recently showed that exposure to PM enhanced the differentiation of naïve T cells into a Th17-like phenotype through an AHR-dependent mechanism (49, 58), contributing to the formation RA. However, the diversity of ligands may lead to different effects on Th17 when AHR activated, when other kinds of ligands, such as, tetrachlorodibenzo-p-dioxin (TCDD), stimulates AHR, it can inhibit Th17 differentiation (59). Therefore, it is speculated that AHR on the initial T cells is a target to prevent the transformation into Th17 cells or that targeting AHR on Th17 cells to control RA has promising therapeutic prospects.

#### 3.1.3 Treg Cells

Treg cells, like Th17 cells, are also a novel type of CD4+ T cell; however, in contrast to Th17 cells, Treg cells secrete anti-inflammatory CKs, such as IL-10 and transforming growth factor-β (TGF-β), which inhibit the progression of autoimmune diseases (53). Treg cells are mainly characterized by cell surface expression of the FoxP3 transcription factor (51). Foxp3 encodes a transcription factor that participates in inflammatory and autoimmune diseases in mice and humans and is specifically expressed in CD4+ Treg cells. Furthermore, retrovirus transfer of the Foxp3 gene induced increased differentiation of naïve T cells into Treg cells. Thus, Foxp3 is a crucial regulatory gene for Treg cell differentiation; in other words, the differentiation, maturation and function of Treg cells are induced by Foxp3 (60). An increasing number of studies have shown that a decreased frequency of FoxP3 T cells (Treg cells) is closely associated with increased disease activity in RA (61). Several studies have shown that Treg cells are significantly reduced in RA patients and CIA model mice (53).

Relationships exist between impairments of Treg and PAHs exposure. A study found substantial increases in PAHs exposure specifically in winter, which was significantly related to the ability and function of Treg cells (62), but the specific mechanism has not been fully characterized.

As receptor of PAHs, AHR is a transcription factor in the cytoplasm of Treg cells and is ligand-dependent (63). In contrast to the low levels of endogenous AHR in naïve T cells, AHR expression was increased in CD4+FoxP3+ cells (63). Recent studies have demonstrated a clear link between Treg cell differentiation and the signaling pathway of AHR, providing a possible mechanism for TCDD mediated immune suppression (30). In support of this conjecture, when AHR was activated with TCDD, Treg cell differentiation was accordingly improved (30). Research has demonstrated a link between Foxp3 and AHR; surprisingly, AHR was found to directly control Foxp3 expression (30) and play a role in Treg cell differentiation. Consistent with this idea, AHR activation by TCDD increased the binding of AHR to the Fox3P promoter and upregulated the expression of the FoxP3 gene to increase the differentiation of Treg cells involved in immunity (30). Conversely, when the mice were exposed to PAHs, Treg cell differentiation was significantly reduced by inhibiting the binding of AHR and the Fox3P promoter to induce the RA (30).

The balance of Treg/Th17 cells is maintained under normal conditions; however, when RA occurs, the balance is broken (53). Experiments have found that AHR affects the differentiation of Th17 cells and Treg cells in opposite ways (30). Although inhibition of Treg cell function and/or hyperfunction of Th17 cells are considered important causes of RA, the specific physiological pathways is known about poorly. Nothing more than, the ability of AHR to selectively modulate the differentiation of Treg cells versus Th17 cells can be exploited, making it a unique target to treat immune diseases.

#### 3.1.4 B Cells

B cells are the only cell type to express immunoglobulin (Ig) on their surface and secrete antibodies after activation. These cells mainly function in humoral immunity, and the humoral response plays a crucial role in the pathogenesis of RA (64, 65). Thus, the activation of B cells plays a key role in the pathogenesis of RA (64). And many studies have shown that the number and activity of B cells are significantly increased in patients with RA (64, 66). One of the main characteristics of RA is the activation of B cells that produce RF and ACPAs. Additionally, some studies have shown that continuous expression of RF increases the activation of B cells and monocytes (65) to aggravate RA. ACPAs enhance NF-κB activity and tumor necrosis factor-α (TNF-α) production by binding to citrullinated Grp78 expressed on macrophages (67). Grp78 protein (72 kD) is a homologous antigen of ACPA, but the mechanism by which the tolerance of B cells is broken remains incompletely understood.

AHR is expressed in B cells cytoplasm in the absence of a ligand (68), which are known targets of PAHs. A study showed that AHR-deficient B cells are less proliferative and less likely to enter the S stage of the cell cycle and remain in the G0/G1 phase of the cell cycle (69), this finding suggests that AHR activated by PAHs might indirectly promote B cell proliferation and activation, aggravating RA and other immune diseases, but enough evidence is needed to support this conjecture. Targeting AHR on B cells also will be a promising treatment in RA.

#### 3.1.5 DCs

DCs are specialized antigen-presenting cells (APCs) that link innate and adaptive immune responses and promote the activation and differentiation of naïve T cells into effector T cells.

DCs are mainly divided into two key distinct functional subsets (conventional myeloid dendritic cells (cDCs) and plasmacytoid dendritic cells (pDCs), induce initial effector differentiation of CD4+ T cells, activate CD8+ T cells, and promote B cell antibody responses, suggesting that DCs may play a crucial role in the initiation of joint inflammation. The interaction between pDCs and T cells (pDCs induce T cell differentiation) induces the autoimmune response in RA, DCs and secreted inflammatory CKs are significantly increased in arthritis model mice (70), and the number of DCs in synovial joint tissues is dramatically increased in RA patients (71). And there was study have shown that DCs are increased in the articular cavity of RA patients (72).

PAHs in the environmental pollutants promote the maturation and differentiation of DCs in an AHR-dependent manner (49). These results imply that PAHs in environmental pollution can induce RA in an AHR-dependent manner by affecting DCs (73).

Additionally, AHR regulates the production of tolerance-related metabolites in a subset of DCs, regulatory DCs (DCregs). This signaling induces the expression of enzymes responsible for tryptophan metabolism, such as indoleamine 2,3-dioxygenases (IDO1 and IDO2). Both IDO1 and IDO2 are immunosuppressive enzymes that participate in kynurenine metabolism to induce the production of Treg cells, which regulate the role of immune suppression by activating AHR (74). Kynurenine inhibits the ability of DCs to initiate T cell responses (73) and contributes to the apoptosis of Th cells, particularly Th1 cells (75). Under normal circumstances, the immune inflammatory and immune tolerance effects of AHR in DCs are balanced; however, in RA patients, a significant bias exists toward the immune inflammatory effect. Currently, strategies targeting immune inflammatory DCs for RA treatment have attracted extensive attention (70), and DCreg therapy for RA is gradually becoming common (76).

#### 3.1.6 Macrophages

Macrophages are also central inflammatory immune cells that play a crucial role in the innate immune function involved in RA and become polarized into different cell phenotypes to mediate inflammatory/immune responses. Studies on the role of macrophages in inflammation have identified two different states of polarization (77), after stimulation, macrophages can mainly become polarized into M1 and M2 macrophages. M1 are pro-inflammatory macrophages, and M2 are anti-inflammatory macrophages. Classically activated macrophages (M1, CD68+CD192+) lead to joint damage with the production of pro-inflammatory CKs, such as TNF-α and IL-1β. Alternatively activated macrophages (M2, CX3CR1+CD163+) produce anti-inflammatory CKs (mainly IL-10 and TGF-β), contributing to tissue remodeling and repair. Recent studies have found that destruction of the balance between M1 and M2 macrophages—that is, a bias toward M1 macrophages, is one of the main reasons for RA (78, 79). The levels of M1 macrophages in RA inflammatory and synovial tissue are increased compared with healthy joints (80).

PAHs are involved in the formation of RA by affecting the maturation and differentiation of macrophages through AHR, which is expressed in both M1 and M2 macrophages cytoplasm, and the expression of AHR is upregulated by PAHs in M1 macrophages, however, down-regulated in M2 macrophages, contributing the increased pro-inflammatory CKs and decreased anti-inflammatory CKs to promote osteoclastogenesis to induce RA (81). Therefore, targeting AHR on different types of macrophages can be developed as a treatment for RA.

#### 3.1.7 NK cells

NK cells also play a crucial role in the pathogenesis of autoimmune diseases (82), they are generally defined by the expression of CD56 and lack of CD3 expression (CD56^+^CD3^-^), and CD56^-^ is another NK cell subtype. These two subtypes differ in maturity, function, and distribution. The main type related to immunoregulatory effects is the CD56^+^ subtype (83), which can produce many CKs after stimulation and exert immunomodulatory functions. Some reports suggest that NK cells markedly expand in the joints and blood of RA patients (82, 84).

Similar to other immune cells, NK cells also have AHR (85), particularly immature CD56^+^ cells (83). After exposure to PAHs, AHR signal pathway is activated, inflammation may occur, and the number of NK cells in inflammatory joints increases (82, 83). AHR activation by PAHs significantly upregulates the expression of the CYP1A1 enzyme (83, 86) in CD56+ NK cells, and CYP1A1 promotes the metabolism of PAHs and alleviates the occurrence of disease. However, PAHs inhibit the function of CYP1A1, leading to the accumulation of PAHs *in vivo* and aggravation of joint inflammation. Therefore, regulation of AHR-targeted CYP1 enzyme activity may be a potential treatment for RA in the future.

#### 3.1.8 OCs

OCs are multinucleated bone cells and responsible for the bone erosion of RA synovial joints, it is the only bone-resorptive cell type (86). OCs are also indispensable for physiological bone remodeling; however, excessive local OCs activity leads to periarticular bone destruction, which is a typical symptom of patients with RA. OCs are mainly derived from mononuclear precursor cells *via* receptor activator of NF-κB ligand (RANKL) and macrophage colony-stimulating factor (M-CSF) signaling. Both RANKL and M-CSF are expressed on NK cells, and NK cells and mononuclear cells are abundant in the inflammatory joints of RA patients. When synovial NK and mononuclear cells are cocultured *in vitro*, mononuclear cells are triggered to differentiate into OCs, and the process relies on RANKL and M-CSF (84). A study found that the number of OCs was increased in RA patients (86).

After stimulation by PAHs, AHR activition plays a crucial role in bone remodeling by altering the interaction between OBs and OCs, and. AHR is expressed in osteocytes, including OBs and OCs (87, 88) There was a study demonstrated that the activation of AHR by B[a]P-mediated (a type of PAHs) stimulates RANKL-induced OCs generation and OCs function in wild-type mice (89) by CYP1A1 enzyme to lead to the formation of arthritis (86, 89).

These findings imply that the AHR, RANKL and CYP1 enzymes may play critical roles in OCs formation. And in the future treatment of RA, AHR-RANKL-CYP1-OCs will be used as an effective therapeutic axis.

#### 3.1.9 OBs

OBs also play an important role in bone remodeling and are involved in bone formation. B cells play a crucial role in OBs dysfunction. In CIA mice, B cells are enriched in subchondral bone marrow and express high levels of genes encoding possible OBs inhibitors such as CCL3 (also called macrophage inflammatory protein 1-α) and TNF-α, which can inhibit OBs differentiation by activating the ERK and NF-κB pathways, CCL3 activates the ERK signaling pathway (90) and TNF-α activates the NF-κB signaling pathway (91). TNF-α is a crucial inflammatory CKs in RA; it not only inhibits OBs bone formation but also triggers OCs bone erosion through the RANK-RANKL pathway by inducing overexpression of Dickkopf-related protein 1 (DKK1), a strong inhibitor of the Wnt signaling pathway for bone synthesis. The regulation of typical Wnt pathways is mainly driven by the production of receptor inhibitors such as DKK1 (92). Additionally, the Wnt pathway plays an important role in the formation of OBs, and increased Wnt signaling pathway activation may lead to decreased OCs formation and bone resorption (93) through upregulating osteoprotegerin (OPG) expression of OBs. The differentiation of OBs is significantly reduced in RA patients (91).

Similar to OCs, AHR also exists in OBs cytoplasm (88), and AHR stimulation has a dose-dependent effect on OBs: overactivation and under-activation inhibit and promote bone formation, respectively. When stimulated by extensive PAHs in the environment, AHR is activated in OBs, causing a series of changes, such as transcriptional activation of CYP1A1 and CYP1B1 (88). CYP1A1 and CYP1B1 polymorphisms alter bone mineral density (BMD) (94, 95) to induce RA disease. A study suggested that women carrying the CYP1B1 gene had increased estrogen catabolism and showed higher urinary estrogen metabolites, the effect may result in relatively low levels of estrogen and low BMD in the lumbar spine and femoral neck of these women (94).

Therefore, for OBs, AHR is the target to regulate the generation of downstream gene CYP1, so as to reduce the catabolism of estrogen to upregulate BMD, which can be a prospective treatment for RA.

## 4 AHR as a Possible Therapeutic Target in RA

AHR is considered an essential factor in immune responses, and many AHR-induced immune mechanisms have been identified, enhancing the understanding of the pathogenesis of immunological inflammatory diseases, including RA, at the molecular level.

Targeting AHR is considered a novel therapeutic target in RA, because it can avoid the long-term use of previous compounds contributing to serious side effects, such as high embryonic mortality (96), recurrent hepatotoxicity (97) and carcinogenicity (98). AHR ligands with fewer side effects and other novel drugs have regarded as potential candidates to treat RA and other autoimmune diseases (**Table 1**). Recently, novel drugs targeting AHR are widely used to treat immune-related diseases, however, AHR ligands have yet to be developed for clinical use. Therefore, we should study the treatment of RA with AHR ligand overall in order to make this therapy more practical in the future.

**Table 1:** Novel agents in RA treatment targeting AHR.

Drugs	Mechanisms of action	Reference
Tetrandrine	promotes the expression of the AHR target gene cytochrome P4501A1(CYP1A1)	(99)
Norisoboldine	upregulates the nuclear translocation of AHR and CYP1A1 expression	(100)
Sinomenine	induces the expression of the AHR-targeted gene CYP1A1, and promotes AHR/Hsp90 dissociation and AHR nuclear translocation	(101)
Human umbilical mesenchymal stem cells (HUMSCs)	increases AHR-target gene and corresponding protein expression	(34)

### 4.1 Traditional Chinese Medicine Monomer Therapy

#### 4.1.1 Tetrandrine

Tetrandrine is a dibenzyl isoquinoline alkaloid isolated from tetrandrine roots and is clinically used to relieve rheumatic pain and joint pain. As an agonist of AHR, tetrandrine ameliorates CIA in mice by inhibiting Th17 cell differentiation and inducing Treg cell formation to restore the balance between Th17 cells and Treg cells, and the primary mechanism of the effect occurs by promoting the expression of the AHR target gene cytochrome P4501A1 (CYP1A1) (99).

#### 4.1.2 Norisoboldine

Norisoboldine (NOR) is the main isoquinoline alkaloid component of polylindera roots that attenuates OCs differentiation to alleviate RA. As an AHR agonist, NOR can stably bind to AHR, upregulate the nuclear translocation of AHR, and enhance the accumulation of the AHR-ARNT complex and AHR-mediated CYP1A1 expression (100) to exert antiarthritic effects. NOR also attenuates the OCs differentiation and bone erosion through activation of AHR and subsequent inhibition of NF-κB pathway and hypoxia-inducible factor (HIF) pathways (100).

#### 4.1.3 Sinomenine

Sinomenine (SIN) is an alkaloid isolated from the root of sinomenine acutum that has been used to treat RA for decades (102). It alleviates arthritis by promoting the production and function of Treg cells in an AHR-dependent manner, inducing the expression of the AHR-targeted gene CYP1A1, and promoting AHR/Hsp90 dissociation and AHR nuclear translocation (101).

### 4.2 Biological Therapy

Our previous studies have shown that human umbilical mesenchymal stem cells (HUMSCs) play a therapeutic role in CIA rats by mediating the interaction between host immunity and gut microbiota through AHR, and this specific mechanism mainly involves increasing AHR target gene and corresponding protein expression (24).

### 4.3 Other Therapeutic Drugs

Various types of dietary phytochemicals, the most typical of which are dietary flavonoids such as quercetin and indigo, may be promising drugs to treat nonalcoholic fatty liver disease (NAFLD) (103) because they inhibit hepatic CD38 and affect AHR (104). Considering its effect on AHR, it can be used as a possible drug to treat RA.

Dietary flavonoids control joint inflammation and reduce arthritis symptoms in both RA patients and CIA animal. Although these substances have powerful anti-inflammatory effects, they are associated with few clinical applications and little scientific evidence regarding their mechanism of action in RA has been reported. Therefore, we should strengthen the research in the area so that the treatment is likely to be promising therapeutic agents for RA in the future.

Although many significant advances have been made in the molecular understanding of biological responses and AHR activation, several crucial questions persist. As Sulire and Kaminski pointed out in their paper, most of the literature on AHR to date has investigated mouse AHR (105). Although mouse and human AHR are interchangeable in most *in vitro* systems, a dramatic difference exists in the binding affinity between the two AHRs to ligands *in vivo*: the mouse AHR binding affinity is 10-fold higher than that of human AHR (106). Thus, the applicability of the results from mouse AHR experiments in human immunity is unclear. A current challenge is that most effects observed in mouse models have yet to be demonstrated in human cells.

## 5 Discussion and Prospects

RA is a chronic autoimmune disease characterized by immune disorder, inflammatory infiltration, articular cartilage damage and joint deformity. The inflammatory infiltration is mainly caused by the increased secretion of inflammatory cells, such as, Th1 (53), Th17 (55), B cells (64, 66), DCs (72), M1 (80), NK cells (82, 84), OCs (86), and corresponding inflammatory CKs, such as, IFN-γ, IL-17, IL-22, TNF-α. Additionly, the decreased Th2 (53), Tregs (53), M2 (79), OBs (91) and IL-10, TGF-β further aggravate the formation of RA.

PAHs in environmental exposure plays a crucial role in the pathogenesis of RA, primarily by influencing the changes in diversity cells and corresponding downstream CKs, and the main pathway of these effects is through the AHR signaling pathway. Interestingly, in these cells, the effects of AHR activation showed an opposite trend. For example, in Th1, Th17, B cells, DCs, M1, NK, and OCs cells, AHR activation had a pathogenic effect on RA (35, 49, 69, 81, 86, 89), however, the activation of Th2 (35), Treg cells (30), DCreg (76), M2 (81) and OBs (88) cells is protective. And the number of these protective cells decreases, the pathogenic effected cells increase, further promoting the occurrence of RA. But, the different types or doses of PAHs mentioned above may produce diverse results in RA. In conclusion, the activation of AHR in RA patients may be either a pathogenic factor or a therapeutic effect, and the specific effect after AHR activation may be related to the type and even the dose of ligands (24).

Although substantial progress has been achieved in the treatment of RA, the current clinical treatment mainly includes nonsteroidal anti-inflammatory drugs, corticosteroids, and disease-modifying antirheumatic drugs (DMARDs). The former two drug classes have an anti-inflammatory analgesic effect soon after administration but do not fundamentally treat RA. DMARDs such as leflunomide (LEF) work slowly but can continuously alleviate disease activity in patients to suppress progressive joint damage and delay the development of RA. Because of their outstanding advantages, DMARDs (particularly new drugs that target AHR) are the main treatments for RA, and their importance is self-evident. In our review, we found that Tetrandrine, NOR, and SIN could play a therapeutic role in RA by targeting AHR. Tetrandrine is to regulate the ratio between Th17 and Treg cells by inhibiting the differentiation of Th17 and promoting the generation of Treg cells through the AHR pathway (99); NOR relieves the differentiation of OCs and erosion of bone by activating AHR (100); SIN mainly plays a role in the treatment of RA by promoting the generation of Treg cells (101). In addition, HUMSCs therapy for RA targets AHR is also a potential treatment (24). Furthermore, the dietary flavonoid in the treatment of liver disease have been reported to relieve joint inflammation and control arthritis symptoms in RA patients and CIA animal models. However, scientific evidence about their mechanism in RA has been poorly studied. The relevant experiments should be carried out and this kind of drugs are likely to be promising therapeutic prescription for RA in the future.

The main purpose of this review was to summarize the possible factors affecting RA and the pathogenesis of RA based on AHR and AHR-targeted drugs for RA treatment, as well as propose novel therapeutic drugs. Although many basic studies have been carried out on PAHs-AHR-RA, clinic studies on influencing the mechanism in RA *via* the AHR signaling pathway are insufficient currently, and further investigations are needed. The development of novel drugs from the laboratory to the clinic through convincing studies are promising probably.

## Data Availability Statement

The original contributions presented in the study are included in the article/supplementary files. Further inquiries can be directed to the corresponding authors.

## Author Contributions

XX and QY contributed equally to this paper. YX and CX designed the manuscript. XX and QY wrote the manuscript. DF, XC, QW, XW and MZ participated in discussions associated with the manuscript. DF, XC and CX revised the manuscript. All the authors read and approved the final manuscript.

## Funding

This study was financially supported by the National Natural Science Foundation of China (Grant number 82073677).

## Conflict of Interest

The authors declare that the research was conducted in the absence of any commercial or financial relationships that could be construed as a potential conflict of interest.

## Publisher’s Note

All claims expressed in this article are solely those of the authors and do not necessarily represent those of their affiliated organizations, or those of the publisher, the editors and the reviewers. Any product that may be evaluated in this article, or claim that may be made by its manufacturer, is not guaranteed or endorsed by the publisher.

## Abbreviations

RA, rheumatoid arthritis; PAHs, polycyclic aromatic hydrocarbons; AHR, aromatic hydrocarbon receptor; AHRR, aryl hydrocarbon receptor repressor; CKs, cytokines; IL, interleukin; TNF-α, tumor necrosis factor-α; RF, rheumatoid factor; ACPA, anti-citrullinated protein antibody; PM, particulate matter; ANY, acenaphthylene; FLT, fluoranthene; B[a]P, benzo [a] pyrene; B[a]A, benzo [a] anthracene; B[b]F, benzo [b] fluoranthene; B[k]F, benzo [k] fluoranthene; DBA, dibenzo (a,h) anthracene; IPY, indeno[1, 2, 3-cd] pyrene; BPE, benzo [ghi] perylene; NAP, naphthalene; ANT, anthracene; PHE, phenanthrene; PYR, pyrene; FLU, fluorene; OPG, osteoprotegerin.

## References

[1] Aaron L Patricia J Torsten M . The World Incidence and Prevalence of Autoimmune Diseases is Increasing. Int J Celiac Dis (2015) 3:151–5. doi: 10.12691/ijcd-3-4-8

[2] Cooper GS Stroehla BC . The Epidemiology of Autoimmune Diseases. Autoimmun Rev (2003) 2:119–225. doi: 10.1016/S1568-9972(03)00006-5 12848952

[3] Cooper GS Bynum MLK Somers EC . Recent Insights in the Epidemiology of Autoimmune Diseases: Improved Prevalence Estimates and Understanding of Clustering of Diseases. J Autoimmun (2009) 33:197–207. doi: 10.1016/j.jaut.2009.09.008 19819109 PMC2783422

[4] Talbot J Peres RS Pinto LG Oliveira RDR Lima KA Donate PB . Smoking-Induced Aggravation of Experimental Arthritis is Dependent of Aryl Hydrocarbon Receptor Activation in Th17 Cells. BioMed Cent (2018) 20:119. doi: 10.1186/s13075-018-1609-9 PMC5994132 29884199

[5] Tamaki A Hayashi H Nakajima H Takii T Katagiri D Miyazawa K . Polycyclic Aromatic Hydrocarbon Increases mRNA Level for Interleukin 1 Beta in Human Fibroblast-Like Synoviocyte Line *via* Aryl Hydrocarbon Receptor. Biol Pharm Bull (2004) 27:407–10. doi: 10.1248/bpb.27.407 14993811

[6] Yang L Yan K Zeng D Lai X Chen X Fang Q . Association of Polycyclic Aromatic Hydrocarbons Metabolites and Risk of Diabetes in Coke Oven Workers. Environ Pollut (2017) 223:305–10. doi: 10.1016/j.envpol.2017.01.027 28131481

[7] Shiue I . Are Urinary Polyaromatic Hydrocarbons Associated With Adult Hypertension, Heart Attack, and Cancer? USA NHANES, 2011–2012. Environ Sci Pollut Res Int (2015) 22:16962–8. doi: 10.1007/s11356-015-4922-8 26111752

[8] Lu DR McDavid AN Kongpachith S Lingampalli N Glanville J Ju CH . T Cell-Dependent Affinity Maturation and Innate Immune Pathways Differentially Drive Autoreactive B Cell Responses in Rheumatoid Arthritis. Arthritis Rheumatol (2018) 70:1732–44. doi: 10.1002/art.40578 PMC6203609 29855173

[9] Ziegelasch M Boman A Martinsson K Thyberg I Jacobs C Nyhall-Wahlin BM . Anti-Cyclic Citrullinated Peptide Antibodies are Associated With Radiographic Damage But Not Disease Activity in Early Rheumatoid Arthritis Diagnosed in 2006–2011. Scand J Rheumatol (2020) 49:434–42. doi: 10.1080/03009742.2020.1771761 32856532

[10] Westerlind H Ronnelid J Hansson M Alfredsson L Mathsson-Alm L Serre G . Anti-Citrullinated Protein Antibody Specificities, Rheumatoid Factor Isotypes, and Incident Cardiovascular Events in Patients With Rheumatoid Arthritis. Arthritis Rheumatol (Hoboken N J) (2020) 72:1658–67. doi: 10.1002/ART.41381 32475073

[11] McInnes IB Schett G . The Pathogenesis of Rheumatoid Arthritis. N Engl J Med (2011) 365:2205–19. doi: 10.1056/NEJMra1004965 22150039

[12] Rahman MH Arslan MI Chen Y Ali S Parvin T Wang LW . Polycyclic Aromatic Hydrocarbon-DNA Adducts Among Rickshaw Drivers in Dhaka City, Bangladesh. Int Arch Occup Environ Health (2003) 76:533–8. doi: 10.1007/s00420-003-0431-z 12827370

[13] Buonanno G Stabile L Morawska L . Personal Exposure to Ultrafine Particles: The Influence of Time-Activity Patterns. Sci Total Environ (2014) 468-469:903–7. doi: 10.1016/j.scitotenv.2013.09.016 24080417

[14] Alex AM Kunkel G Sayles H Arcos JDF Mikuls TR Kerr GS . Exposure to Ambient Air Pollution and Autoantibody Status in Rheumatoid Arthritis. Clin Rheumatol (2020) 39:761–8. doi: 10.1007/s10067-019-04813-w 31729679

[15] Chang K Hsu CC Muo CH Hsu CY Liu HC Kao CH . Air Pollution Exposure Increases the Risk of Rheumatoid Arthritis: A Longitudinal and Nationwide Study. Environ Int (2016) 94:495–9. doi: 10.1016/j.envint.2016.06.008 27302847

[16] Shepherd A Mullins JT . Exposure to Traffic Pollution and Increased Risk of Rheumatoid Arthritis. Environ Health Perspect (2009) 117:1065–9. doi: 10.1289/ehp.0800503 PMC2717131 19654914

[17] Shepherd A Mullins JT . Arthritis Diagnosis and Early-Life Exposure to Air Pollution. Environ Pollut (2019) 253:1030–7. doi: 10.1016/j.envpol.2019.07.054 31434180

[18] Hart JE Laden F Puett RC Costenbader KH Karlson EW . Association Between Exposure to Ambient Air Pollution and Rheumatoid Arthritis in Adults. Int J Environ Res Public Health (2019) 16:1227. doi: 10.3390/ijerph16071227 PMC6480037 30959862

[19] Vasudha B Ki-Hyun K . Review of PAH Contamination in Food Products and Their Health Hazards. Environ Int (2015) 84:26–38. doi: 10.1016/j.envint.2015.06.016 26203892

[20] Chen C Min Y Li X Chen D Shen J Zhang D . Mutagenicity Risk Prediction of PAH and Derivative Mixtures by in Silico Simulations Oriented From CYP Compound I-Mediated Metabolic Activation. Sci Total Environ (2021) 787:147596–6. doi: 10.1016/J.SCITOTENV.2021.147596 33991922

[21] Tsutomu S Peter GF . Inhibition of Human Cytochrome P450 1A1-, 1A2-, and 1B1-Mediated Activation of Procarcinogens to Genotoxic Metabolites by Polycyclic Aromatic Hydrocarbons. Chem Res Toxicol (2006) 19:288–94. doi: 10.1021/tx050291v 16485905

[22] Shimada T Murajama N Tanaka K Takenaka S Imai Y Hopkins NE . Interaction of Polycyclic Aromatic Hydrocarbons With Human Cytochrome P450 1B1 in Inhibiting Catalytic Activity. Chem Res Toxicol (2008) 21:2313–23. doi: 10.1021/tx8002998 PMC2772130 19548353

[23] Li J Li X Xi Y Fan H Fan D Xi X . Subgroup Analysis of the Relationship Between Polycyclic Aromatic Hydrocarbons and Rheumatoid Arthritis: Data From the National Health and Nutrition Examination Survey, 2003–2014. Sci Total Environ (2021) 775:145841. doi: 10.1016/J.SCITOTENV.2021.145841 33621881

[24] Li X Fan H Li J Fan D Lu X Lv S . Study on the Correlation Between Polycyclic Aromatic Hydrocarbon Exposure and Rheumatoid Arthritis. Chin J Gerontol (2020) 39:67–72. doi: 10.3760/cma.j.issn.0254-9026.2020.01.013

[25] Burbach KM Poland A Bradfield CA . Cloning of the Ah-Receptor cDNA Reveals a Distinctive Ligand-Activated Transcription Factor. Proc Natl Acad Sci USA (1992) 89:8185–9. doi: 10.1073/pnas.89.17.8185 PMC49882 1325649

[26] Fukunaga BN Probst MR Reisz-Porszasz S Hankinson O . Identification of Functional Domains of the Aryl Hydrocarbon Receptor. J Biol Chem (1995) 270:29270–8. doi: 10.1074/jbc.270.49.29270 7493958

[27] Crews ST F CM . Remembrance of Things PAS: Regulation of Development by bHLH-PAS Proteins. Curr Opin Genet Dev (1999) 9:580–7. doi: 10.1016/s0959-437x(99)00003-9 10508688

[28] Gu YZ Hogenesch JB Bradfield CA . The PAS Superfamily: Sensors of Environmental and Developmental Signals. Annu Rev Pharmacol (2000) 40:519–61. doi: 10.1146/annurev.pharmtox.40.1.519 10836146

[29] Peggy P Ho LS . The Aryl Hydrocarbon Receptor: A Regulator of Th17 and Treg Cell Development in Disease. Cell Res (2008) 18:605–8. doi: 10.1038/cr.2008.63 18516065

[30] Quintana FJ Basso AS Iglesias AH Korn T Farez MF Bettelli E . Control of T(reg) and T(H)17 Cell Differentiation by the Aryl Hydrocarbon Receptor. Nature (2008) 453:65–71. doi: 10.1038/nature06880 18362915

[31] Veldhoen M Hirota K Westendorf AM Buer J Dumoutier L Renauld J . The Aryl Hydrocarbon Receptor Links TH17-Cell-Mediated Autoimmunity to Environmental Toxins. Nature (2008) 453:106–9. doi: 10.1038/nature06881 18362914

[32] Mascanfroni ID Takenaka MC Yeste A Patel B Wu Y Kenison JE . Metabolic Control of Type 1 Regulatory T Cell Differentiation by AHR and HIF1-α. Nat Med (2015) 21:638–46. doi: 10.1038/nm.3868 PMC4476246 26005855

[33] Ambrosio LF Insfran C Volpini X Rodriguez EA Serra HM Quintana FJ . Role of Aryl Hydrocarbon Receptor (AhR) in the Regulation of Immunity and Immunopathology During Trypanosoma Cruzi Infection. Front Immunol (2019) 10:631. doi: 10.3389/fimmu.2019.00631 30984194 PMC6450169

[34] Li X Lu C Fan D Lu X Xia Y Zhao H . Human Umbilical Mesenchymal Stem Cells Display Therapeutic Potential in Rheumatoid Arthritis by Regulating Interactions Between Immunity and Gut Microbiota *via* the Aryl Hydrocarbon Receptor. Front Cell Dev Biol (2020) 8:131. doi: 10.3389/fcell.2020.00131 32232043 PMC7082776

[35] Nakahama T Kimura A Nguyen NT Chinen I Hanieh H Nohara K . Aryl Hydrocarbon Receptor Deficiency in T Cells Suppresses the Development of Collagen-Induced Arthritis. Proc Natl Acad Sci USA (2011) 108:14222–7. doi: 10.1073/pnas.1111786108 PMC3161527 21825138

[36] Negishi T Kato Y Ooneda O Mimura J Takada T Mochizuki H . Effects of Aryl Hydrocarbon Receptor Signaling on the Modulation of TH1/TH2 Balance. J Immunol (Baltimore Md 1950) (2005) 175:7348–56. doi: 10.4049/jimmunol.175.11.7348 16301641

[37] McLane KE Whitlock JP . DNA Sequence Requirements for Ah Receptor/Arnt Recognition Determined by *In Vitro* Transcription. Receptor (1994) 4:209–22. 7894337

[38] Shen ES Whitlock JJ . Protein-DNA Interactions at a Dioxin-Responsive Enhancer. Mutational Analysis of the DNA-Binding Site for the Liganded Ah Receptor. J Biol Chem (1992) 267:6815–9. doi: 10.1016/S0021-9258(19)50499-9 1313023

[39] Zanger UM Schwab M . Cytochrome P450 Enzymes in Drug Metabolism: Regulation of Gene Expression, Enzyme Activities, and Impact of Genetic Variation. Pharmacol Ther (2013) 138:130–41. doi: 10.1016/j.pharmthera.2012.12.007 23333322

[40] Gutierrez-Vazquez C Quintana FJ . Regulation of the Immune Response by the Aryl Hydrocarbon Receptor. Immunity (2018) 48:19–33. doi: 10.1016/j.immuni.2017.12.012 29343438 PMC5777317

[41] Shimada T Murayama N Yamazaki H Tanaka K Takenaka S Komori M . Metabolic Activation of Polycyclic Aromatic Hydrocarbons and Aryl and Heterocyclic Amines by Human Cytochromes P450 2A13 and 2A6. Chem Res Toxicol (2013) 26:529–37. doi: 10.1021/tx3004906 PMC3713097 23432465

[42] Ikuta T Tachibana T Watanabe J Yoshida M Yoneda Y Kawajiri K . Nuclear Localization and Export Signals of the Human Aryl Hydrocarbon Receptor. J Biol Chem (1998) 273:2895–904. doi: 10.1074/jbc.273.5.2895 9446600

[43] Ikuta T Eguchi H Tachibana T Yoneda Y Kawajiri K . Nucleocytoplasmic Shuttling of the Aryl Hydrocarbon Receptor. Jpn Biochem Soc (2008) 127:503–9. doi: 10.1093/oxfordjournals.jbchem.a022633 10731723

[44] Davarinos NA Pollenz RS . Aryl Hydrocarbon Receptor Imported Into the Nucleus Following Ligand Binding Is Rapidly Degraded *via* the Cytosplasmic Proteasome Following Nuclear Export. J Biol Chem (1999) 274:28708–15. doi: 10.1074/jbc.274.40.28708 10497241

[45] Ma Q Baldwin KT . 2,3,7,8-Tetrachlorodibenzo-P-Dioxin-Induced Degradation of Aryl Hydrocarbon Receptor (AhR) by the Ubiquitin-Proteasome Pathway. Role of the Transcription Activation and DNA Binding of AhR. J Biol Chem (2000) 275:8432–8. doi: 10.1074/jbc.275.12.8432 10722677

[46] Mimura J Ema M Sogawa K Fujii-Kuriyama Y . Identification of a Novel Mechanism of Regulation of Ah (Dioxin) Receptor Function. Genes Development (1999) 13:20–5. doi: 10.1101/gad.13.1.20 PMC316371 9887096

[47] Shimada T Guengerich FP . Xenobiotic-Metabolizing Enzymes Involved in Activation and Detoxification of Carcinogenic Polycyclic Aromatic Hydrocarbons. Jpn Soc Study Xenobiotics (2006) 21:257–76. doi: 10.2133/dmpk.21.257 16946553

[48] Cavret S Feidt C . Intestinal Metabolism of PAH: *In Vitro* Demonstration and Study of its Impact on PAH Transfer Through the Intestinal Epithelium. Environ Res (2005) 98:22–32. doi: 10.1016/j.envres.2004.10.010 15721880

[49] O'Driscoll CA Gallo ME Hoffmann EJ Fechner JH Schauer JJ Bradfield CA . Polycyclic Aromatic Hydrocarbons (PAHs) Present in Ambient Urban Dust Drive Proinflammatory T Cell and Dendritic Cell Responses *via* the Aryl Hydrocarbon Receptor (AHR) *In Vitro* . PloS One (2018) 13:1–19. doi: 10.1371/journal.pone.0209690 PMC6303068 30576387

[50] Lauer LF Parvez F Factor-Litvak P Liu X Santella RM Islam T . Changes in Human Peripheral Blood Mononuclear Cell (HPBMC) Populations and T-Cell Subsets Associated With Arsenic and Polycyclic Aromatic Hydrocarbon Exposures in a Bangladesh Cohort. PloS One (2019) 14:e0220451. doi: 10.1371/journal.pone.0220451 31365547 PMC6668812

[51] Moser M Leo O . Key Concepts in Immunology. Vaccine (2010) 28:C2–C13. doi: 10.1016/j.vaccine.2010.07.022 20713253

[52] Wu S Zhou Y Liu S Zhang H Luo H Zuo X . Regulatory Effect of Nicotine on the Differentiation of Th1, Th2 and Th17 Lymphocyte Subsets in Patients With Rheumatoid Arthritis. Eur J Pharmacol (2018) 831:38–45. doi: 10.1016/j.ejphar.2018.04.028 29715455

[53] Li C Zhang J Wang W Wang H Zhang Y Zhang Z . Data on Arsenic Trioxide Modulates Treg/Th17/Th1/Th2 Cells in Treatment-Naïve Rheumatoid Arthritis Patients and Collagen-Induced Arthritis Model Mice. Data Brief (2019) 27:104615. doi: 10.1016/j.dib.2019.104615 31871961 PMC6915806

[54] Ivanov II McKenzie BS Zhou L Tadokoro CE Lepelley A Lafaille JJ . The Orphan Nuclear Receptor RORgammat Directs the Differentiation Program of Proinflammatory IL-17+ T Helper Cells. Cell (2006) 126:1121–33. doi: 10.1016/j.cell.2006.07.035 16990136

[55] Van Hamburg JP Asmawidjaja PS Davelaar N Mus AMC Colin EM Hazes JMW . Th17 Cells, But Not Th1 Cells, From Patients With Early Rheumatoid Arthritis are Potent Inducers of Matrix Metalloproteinases and Proinflammatory Cytokines Upon Synovial Fibroblast Interaction, Including Autocrine Interleukin-17A Production. Arthritis Rheum (2011) 63:73–83. doi: 10.1002/art.30093 20954258

[56] Schiering C Vonk A Das S Stockinger B Wincent E . Cytochrome P4501-Inhibiting Chemicals Amplify Aryl Hydrocarbon Receptor Activation and IL-22 Production in T Helper 17 Cells. Biochem Pharmacol (2018) 151:47–58. doi: 10.1016/j.bcp.2018.02.031 29501585

[57] Kimura A Naka T Nohara K Fujii-Kuriyama Y Kishimoto T . Aryl Hydrocarbon Receptor Regulates Stat1 Activation and Participates in the Development of Th17 Cells. Proc Natl Acad Sci U S A (2008) 105:9721–6. doi: 10.1073/pnas.0804231105 PMC2474493 18607004

[58] Castaneda AR Pinkerton KE Bein KJ Magana-Mendez A Yang HT Ashwood P . Ambient Particulate Matter Activates the Aryl Hydrocarbon Receptor in Dendritic Cells and Enhances Th17 Polarization. Toxicol Lett (2018) 292:85–96. doi: 10.1016/j.toxlet.2018.04.020 29689377 PMC5971007

[59] Suo X Li H . Experimental Study of 2,3,7, 8-Tetrachlorodiphenyl-P-Dioxin Regulating the Balance of Helper T Cells 17/ Regulatory T Cells in the Treatment of Rheumatoid Arthritis. Chin J Rheumatol (2016) 20:34–9. doi: 10.3760/cma.j.issn.1007-7480.2016.01.008

[60] Hori S Nomura T Sakaguchi S . Control of Regulatory T Cell Development by the Transcription Factor Foxp3. Science (2003) 299:1057–61. doi: 10.1126/science.1079490 12522256

[61] Sun H Gao W Pan W Zhang Q Wang G Feng D . Tim3+ Foxp3 + Treg Cells Are Potent Inhibitors of Effector T Cells and Are Suppressed in Rheumatoid Arthritis. Inflammation (2017) 40:1342–50. doi: 10.1007/s10753-017-0577-6 28478516

[62] Yao Y Wang D Ma H Li C Chang X Low P . The Impact on T-Regulatory Cell Related Immune Responses in Rural Women Exposed to Polycyclic Aromatic Hydrocarbons (PAHs) in Household Air Pollution in Gansu, China: A Pilot Investigation. Environ Res (2019) 173:306–17. doi: 10.1016/j.envres.2019.03.053 30951957

[63] Funatake CJ Marshall NB Steppan LB Mourich DV Kerkvliet NI . Cutting Edge: Activation of the Aryl Hydrocarbon Receptor by 2,3,7,8-Tetrachlorodibenzo-P-Dioxin Generates a Population of CD4+ CD25+ Cells With Characteristics of Regulatory T Cells. J Immunol (Baltimore Md 1950) (2005) 175:4184–8. doi: 10.4049/jimmunol.175.7.4184 16177056

[64] Chen C Hu FL Liu HJ Xu LL Li YN Li ZG . [Myeloid-Derived Suppressor Cells Promoted Autologous B Cell Proliferation in Rheumatoid Arthritis]. Beijing Da Xue Xue Bao Yi Xue Ban (2017) 49:819–23. doi: 10.3969/j.issn.1671-167X.2017.05.013 29045962

[65] Davis JM Crowson CS Knutson KL Achenbach SJ Strausbauch MA Therneau TM . Longitudinal Relationships Between Rheumatoid Factor and Cytokine Expression by Immunostimulated Peripheral Blood Lymphocytes From Patients With Rheumatoid Arthritis: New Insights Into B-Cell Activation. Clin Immunol (2020) 211:108342. doi: 10.1016/j.clim.2020.108342 31926330 PMC7045286

[66] Magalhaes R Stiehl P Morawietz L Berek C Krenn V . Morphological and Molecular Pathology of the B Cell Response in Synovitis of Rheumatoid Arthritis. Virchows Arch (2002) 441:415–27. doi: 10.1007/s00428-002-0702-1 12447670

[67] Lu MC Lai NS Yu HC Huang HB Hsieh SC Yu CL . Anti-Citrullinated Protein Antibodies Bind Surface-Expressed Citrullinated Grp78 on Monocyte/Macrophages and Stimulate Tumor Necrosis Factor Alpha Production. Arthritis Rheum (2010) 62:213–1223. doi: 10.1002/art.27386 20213805

[68] Kovalova N Nault R Crawford R Zacharewski TR Kaminski NE . Comparative Analysis of TCDD-Induced AhR-Mediated Gene Expression in Human, Mouse and Rat Primary B Cells. Toxicol Appl Pharm (2017) 316:95–106. doi: 10.1016/j.taap.2016.11.009 PMC5292778 27913140

[69] Villa M Gialitakis M Tolaini M Ahlfors H Henderson CJ Wolf CR . Aryl Hydrocarbon Receptor is Required for Optimal B-Cell Proliferation. EMBO J (2017) 36:116–28. doi: 10.15252/embj.201695027 PMC5210087 27875245

[70] Chuang CH Cheng YC Lin SC Lehman CW Wang SP Chen DY . Atractylodin Suppresses Dendritic Cell Maturation and Ameliorates Collagen-Induced Arthritis in a Mouse Model. J Agric Food Chem (2019) 67:6773–84. doi: 10.1021/acs.jafc.9b01163 31154759

[71] Pan F Xiang H Yan J Hong L Zhang L Liu Y . Dendritic Cells From Rheumatoid Arthritis Patient Peripheral Blood Induce Th17 Cell Differentiation *via* miR-363/Integrin αv/TGF-β Axis. Scand J Immunol (2017) 85:441–9. doi: 10.1111/sji.12550 28376277

[72] Zvaifler NJ Steinman RM Kaplan G Lau LL Rivelis M . Identification of Immunostimulatory Dendritic Cells in the Synovial Effusions of Patients With Rheumatoid Arthritis. J Clin Invest (1985) 76:789–800. doi: 10.1172/JCI112036 3875632 PMC423902

[73] Nguyen NT Kimura A Nakahama T Chinen I Masuda K Nohara K . Aryl Hydrocarbon Receptor Negatively Regulates Dendritic Cell Immunogenicity *via* a Kynurenine-Dependent Mechanism. Proc Natl Acad Sci USA (2010) 107:19961–6. doi: 10.1073/pnas.1014465107 PMC2993339 21041655

[74] Vogel CFA Goth SR Dong B Pessah IN Matsumura F . Aryl Hydrocarbon Receptor Signaling Mediates Expression of Indoleamine 2,3-Dioxygenase. Biochem Biophys Res Commun (2008) 375:331–5. doi: 10.1016/j.bbrc.2008.07.156 PMC2583959 18694728

[75] Mellor AL Baban B Chandler P Marshall B Jhaver K Hansen A . Cutting Edge: Induced Indoleamine 2,3 Dioxygenase Expression in Dendritic Cell Subsets Suppresses T Cell Clonal Expansion. J Immunol (Baltimore Md 1950) (2003) 171:1652–5. doi: 10.4049/jimmunol.171.4.1652 12902462

[76] Lee LE Sul JH Shin JM Shin S Lee JA Kim HK . Reactive Oxygen Species-Responsive Dendritic Cell-Derived Exosomes for Rheumatoid Arthritis. Acta Biomater (2021) 128:462–73. doi: 10.1016/J.ACTBIO.2021.04.026 33878476

[77] Mohammadi A Blesso CN Barreto GE Banach M Majeed M Sahebkar A . Macrophage Plasticity, Polarization and Function in Response to Curcumin, a Diet-Derived Polyphenol, as an Immunomodulatory Agent. J Nutr Biochem (2019) 66:1–16. doi: 10.1016/j.jnutbio.2018.12.005 30660832

[78] Patel U Rajasingh S Samanta S Cao T Dawn B Rajasingh J . Macrophage Polarization in Response to Epigenetic Modifiers During Infection and Inflammation. Drug Discov Today (2017) 22:186–93. doi: 10.1016/j.drudis.2016.08.006 PMC5226865 27554801

[79] Alberto M . Macrophage Diversity and Polarization: *In Vivo* Veritas. Blood (2006) 108:408–9. doi: 10.1182/blood-2006-05-019430

[80] Mulherin D Fitzgerald O Bresnihan B . Synovial Tissue Macrophage Populations and Articular Damage in Rheumatoid Arthritis. Arthritis Rheum (1996) 39:115–24. doi: 10.1002/art.1780390116 8546720

[81] Fukui S Iwamoto N Takatani A Igawa T Shimizu T Umeda M . M1 and M2 Monocytes in Rheumatoid Arthritis: A Contribution of Imbalance of M1/M2 Monocytes to Osteoclastogenesis. Front Immunol (2017) 8:1958. doi: 10.3389/fimmu.2017.01958 29375576 PMC5766997

[82] Dalbeth N Callan MFC . A Subset of Natural Killer Cells is Greatly Expanded Within Inflamed Joints. Arthritis Rheum (2002) 46:1763–72. doi: 10.1002/art.10410 12124859

[83] Moreno-Nieve UY Mundy DC Shin JH Tam K Sunwoo JB . The Aryl Hydrocarbon Receptor Modulates the Function of Human CD56bright NK Cells. Eur J Immunol (2018) 48:771–6. doi: 10.1002/eji.201747289 PMC6658132 29336030

[84] Soderstrom K Stein E Colmenero P Purath U Muller-Ladner U Matos CT . Natural Killer Cells Trigger Osteoclastogenesis and Bone Destruction in Arthritis. Proc Natl Acad Sci USA (2010) 107:13028–33. doi: 10.1073/pnas.1000546107 PMC2919936 20615964

[85] Wagage S John B Krock BL Hall AO Randall LM Karp CL . The Aryl Hydrocarbon Receptor Promotes IL-10 Production by NK Cells. J Immunol (Baltimore Md 1950) (2014) 192:1661–170. doi: 10.4049/jimmunol.1300497 PMC3955958 24403534

[86] Van Raemdonck K Umar S Palasiewicz K Volkov S Volin MV Arami S . CCL21/CCR7 Signaling in Macrophages Promotes Joint Inflammation and Th17-Mediated Osteoclast Formation in Rheumatoid Arthritis. Cell Mol Life Sci (2020) 77:1387–99. doi: 10.1007/s00018-019-03235-w PMC10040247 31342120

[87] Ilvesaro J Pohjanvirta R Tuomisto J Viluksela M Tuukkanen J . Bone Resorption by Aryl Hydrocarbon Receptor-Expressing Osteoclasts is Not Disturbed by TCDD in Short-Term Cultures. Life Sci (2005) 77:1351–66. doi: 10.1016/j.lfs.2005.01.027 15913656

[88] Miki Y Hata S Ono K Suzuki T Ito K Kumamoto H . Roles of Aryl Hydrocarbon Receptor in Aromatase-Dependent Cell Proliferation in Human Osteoblasts. Int J Mol Sci (2017) 18:1–12. doi: 10.3390/ijms18102159 PMC5666840 29039776

[89] Iqbal J Sun L Cao J Yuen T Lu P Bab I . Smoke Carcinogens Cause Bone Loss Through the Aryl Hydrocarbon Receptor and Induction of Cyp1 Enzymes. Proc Natl Acad Sci USA (2013) 110:11115–20. doi: 10.1073/pnas.1220919110 PMC3704019 23776235

[90] Vallet S Pozzi S Patel K Vaghela N Fulciniti MT Veiby P . A Novel Role for CCL3 (MIP-1α) in Myeloma-Induced Bone Disease *via* Osteocalcin Downregulation and Inhibition of Osteoblast Function. Leukemia (2011) 25:1174–81. doi: 10.1038/leu.2011.43 PMC4142423 21403648

[91] Yao Z Li Y Yin X Dong Y Xing L Boyce BF . NF-κB RelB Negatively Regulates Osteoblast Differentiation and Bone Formation. J Bone Miner Res (2014) 29:866–77. doi: 10.1002/jbmr.2108 PMC3961566 24115294

[92] Rossini M Gatti D Adami S . Involvement of WNT/β-Catenin Signaling in the Treatment of Osteoporosis. Calcif Tissue Int (2013) 93:121–32. doi: 10.1007/s00223-013-9749-z 23748710

[93] Spencer GJ Utting JC Etheridge SL Arnett TR Genever PG . Wnt Signalling in Osteoblasts Regulates Expression of the Receptor Activator of NFkappaB Ligand and Inhibits Osteoclastogenesis *In Vitro* . J Cell Sci (2006) 119:1283–96. doi: 10.1242/jcs.02883 16522681

[94] Napoli N Rini GB Serber D Giri T Yarramaneni J Bucchieri S . The Val432Leu Polymorphism of the CYP1B1 Gene is Associated With Differences in Estrogen Metabolism and Bone Density. Bone (2009) 44:442–8. doi: 10.1016/j.bone.2008.09.018 PMC3966713 18977467

[95] Quan J Yahata T Tamura N Nagata H Tanaka K . Relationship Between Single Nucleotide Polymorphisms in CYP1A1 and CYP1B1 Genes and the Bone Mineral Density and Serum Lipid Profiles in Postmenopausal Japanese Women Taking Hormone Therapy. Menopause (2009) 16:171–6. doi: 10.1097/gme.0b013e31817ed24f 18779756

[96] Jonsson ME Mattsson A Shaik S Brunstrom B . Toxicity and Cytochrome P450 1a mRNA Induction by 6-Formylindolo[3,2- B ]Carbazole (FICZ) in Chicken and Japanese Quail Embryos. Comp Biochem Physiol Part C (2016) 179:125–36. doi: 10.1016/j.cbpc.2015.09.014 26456929

[97] Pierre S Chevallier A Teixeira-Clerc F Ambolet-Camoit A Bui L Bats AS . Aryl Hydrocarbon Receptor-Dependent Induction of Liver Fibrosis by Dioxin. Toxicol Sci (2014) 137:114–24. doi: 10.1093/toxsci/kft236 24154488

[98] Kalkhof S Dautel F Loguercio S Baumann S Trump S Jungnickel H . Pathway and Time-Resolved Benzo[a]Pyrene Toxicity on Hepa1c1c7 Cells at Toxic and Subtoxic Exposure. J Proteome Res (2015) 14:164–82. doi: 10.1021/pr500957t 25362887

[99] Yuan X Tong B Dou Y Wu X Wei Z Dai Y . Tetrandrine Ameliorates Collagen-Induced Arthritis in Mice by Restoring the Balance Between Th17 and Treg Cells *via* the Aryl Hydrocarbon Receptor. Biochem Pharmacol (2016) 101:87–99. doi: 10.1016/j.bcp.2015.11.025 26640276

[100] Wei Z Lv Q Xia Y Yue M Shi C Xia Y . Norisoboldine, an Anti-Arthritis Alkaloid Isolated From Radix Linderae, Attenuates Osteoclast Differentiation and Inflammatory Bone Erosion in an Aryl Hydrocarbon Receptor-Dependent Manner. Int J Biol Sci (2015) 11:1113–26. doi: 10.7150/ijbs.12152 PMC4515821 26221077

[101] Tong B Yuan X Dou Y Wu X Wang Y Xia Y . Sinomenine Induces the Generation of Intestinal Treg Cells and Attenuates Arthritis *via* Activation of Aryl Hydrocarbon Receptor. Lab Invest J Tech Methods Pathol (2016) 96:1076–86. doi: 10.1038/labinvest.2016.86 27617398

[102] Feng ZT Yang T Hou XQ Wu HY Feng JF Ou BJ . Sinomenine Mitigates Collagen-Induced Arthritis Mice by Inhibiting Angiogenesis. BioMed Pharmacother (2019) 113:1–7. doi: 10.1016/j.biopha.2019.108759 30856539

[103] Yang H Yang T Heng C Zhou Y Jiang Z Qian X . Quercetin Improves Nonalcoholic Fatty Liver by Ameliorating Inflammation, Oxidative Stress, and Lipid Metabolism in Db/Db Mice. Phytother Res (2019) 33:3140–52. doi: 10.1002/ptr.6486 31452288

[104] Bock KW . Modulation of Aryl Hydrocarbon Receptor (AHR) and the NAD + -Consuming Enzyme CD38: Searches of Therapeutic Options for Nonalcoholic Fatty Liver Disease (NAFLD). Biochem Pharmacol (2020) 175:113905. doi: 10.1016/j.bcp.2020.113905 32169417

[105] Sulentic CE Kaminski NE . The Long Winding Road Toward Understanding the Molecular Mechanisms for B-Cell Suppression by 2,3,7,8-Tetrachlorodibenzo-P-Dioxin. Toxicol Sci (2011) 120 Suppl 1:171–91. doi: 10.1093/toxsci/kfq324 PMC3043085 20952503

[106] Ema M Ohe N Suzuki M Mimura J Sogawa K Ikawa S . Dioxin Binding Activities of Polymorphic Forms of Mouse and Human Aryl Hydrocarb Receptors. J Biol Chem (1994) 269:27337–43. doi: 10.1016/S0021-9258(18)46990-6 7961644

